# Expectancy in placebo-controlled trials of psychedelics: if so, so what?

**DOI:** 10.1007/s00213-022-06221-6

**Published:** 2022-09-05

**Authors:** Matt Butler, Luke Jelen, James Rucker

**Affiliations:** Institute of Psychiatry, Psychology, and Neuroscience, King’s College London, London, UK

**Keywords:** Psilocybin, Psychoactive medication, Clinical trials, Expectancy, Placebo

## Abstract

Modern psychedelic research remains in an early phase, and the eventual introduction of psychedelics into clinical practice remains in doubt. In this piece, we discuss the role of blinding and expectancy in psychedelic trials, and place this in a broader historical and contemporary context of blinding in trials across the rest of healthcare. We suggest that premature and uncritical promotion (‘hype’) of psychedelics as medicines is not only misleading, but also directly influences participant expectancy in ongoing psychedelic trials. We argue that although psychedelic trials are likely to significantly overestimate treatment effects by design due to unblinding and expectancy effects, this is not a unique situation. Placebo-controlled RCTs are not a perfect fit for all therapeutics, and problems in blinding should not automatically disqualify medications from licencing decisions. We suggest that simple practical measures may be (and indeed already are) taken in psychedelic trials to partially mitigate the effects of expectancy and unblinding, such as independent raters and active placebos. We briefly suggest other alternative trial methodologies which could be used to bolster RCT results, such as naturalistic studies. We conclude that the results of contemporary placebo-controlled RCTs of psychedelics should neither be dismissed due to imperfections in design, nor should early data be taken as firm evidence of effectiveness.

## Introduction

During the 1950s and 1960s, psychedelics (including lysergic acid dimethylamine [LSD] and psilocybin) were extensively investigated for a range of psychiatric disorders. These early studies invariably had methodological shortcomings by current standards (e.g. anecdotal evidence, inadequate assessment procedures, lack of controls) (J. J. H. Rucker et al. 2018). Nevertheless, systematic reviews suggest that when administered in combination with psychological therapy, psychedelics were associated with impressive improvement rates in depression, anxiety and alcohol dependence (J. J. H. Rucker et al. 2016; Weston et al. 2020; Krebs and Johansen 2012). Following the decision to place psychedelics in Schedule I of the 1971 UN Convention on Psychotropic Substances, research dwindled until the turn of the millennium (Nutt and Carhart-Harris 2021).

The last two decades has seen a resurgence of psychedelic research, and their potential medical use is now being carefully re-examined in modern clinical trials. Although there is some evidence indicating their possible efficacy in early trials of several psychiatric and neurological conditions (Andersen et al. 2021), there are currently no data to indicate their effectiveness (that is the degree to which they are beneficial in ‘real world’ settings). The possibility of their introduction to future routine clinical practice remains in doubt.

Modern psychedelic research has emerged within an environment of lingering cultural stigma surrounding psychedelics and their use, as well as premature and unjustified hype about their medical efficacy (Noorani and Martell 2021). Concerns have been raised by many in the field that psychedelics are uncritically promoted to patients and the public (Yaden et al. 2021). Well-designed modern trials are attempting to address both issues of stigma and hype (Muthukumaraswamy et al. 2022) and are attempting to avoid the pitfalls of trials undertaken prior to 1971, which were often suboptimal in design and generally not subject to rigorous oversight (J. J. H. Rucker et al. 2018).

Psychedelics used in clinical trials, ranging from the short acting 5-methoxy-N,N-dimethyltryptamine (5-MEO-DMT), to longer acting psilocybin, are united in eliciting recognisable subjective effects (‘trips’) at common doses (Ermakova et al. 2021; Nichols 2016). In other words, psychedelics are psychoactive, a property they share with several other medications currently under investigation for neuropsychiatric disorders, such as 3,4-methylenedioxymethamphetamine (MDMA), cannabis and (es)ketamine, as well as several licenced medications, such as benzodiazepines, gabapentinoids (gabapentin, pregabalin), opiates and stimulants (e.g. methylphenidate, dextroamphetamine).

The subjective effects of active psychedelic doses are thought to be relatively easy to discern by the average participant in modern clinical trials (although it is also possible to have a subjective trip on a placebo (J. A. Olson et al. 2020)). Due to this difficulty in blinding subjects to treatment allocation, concerns have been raised about the interpretation of efficacy from clinical trials (Burke and Blumberger 2021; Muthukumaraswamy et al. 2021). There have been several well-written pieces which situate this problem and suggest remedial solutions, such as monitoring the expectancy of participants enrolled in psychedelic clinical trials or altering trial designs to include more conditions than standard two-way comparisons (Muthukumaraswamy et al. 2021; Aday et al. 2021).

## Blinding and expectancy

Consensus agreement and guidelines conclude that the highest quality of evidence for assessing efficacy of therapeutics comes from randomised controlled trials (RCTs) (Muthukumaraswamy et al. 2021). Properly-designed RCTs aim to mitigate as many sources of bias as possible, including methodological features such as blinding of participants and assessors to treatment allocation in an attempt to mitigate expectancy effects (Probst et al. 2016).

The importance of blinding in clinical trials has been recognised for over a century. The first practitioner to utilise blinding was probably the psychiatrist WHR Rivers, who in 1906 self-administered small amounts of either an alcoholic or non-alcoholic drink which had been prepared by a colleague to a standard recipe which entirely masked the flavour of the former. He aimed to assess the effects of these drinks on muscle fatigability and recognised that previous studies on the same topic likely over-estimated effects due to the participants being aware of whether or not they had tasted alcohol (Rivers and Webber 1908)*.*

Since the mid-twentieth century, blinding has been standard for clinical trials of medication, mostly due to the overwhelming evidence that inadequate blinding tends to over-estimate treatment effects (Jüni et al. 2001). Double-blind trials, in which neither the participant nor the trial practitioner is aware of treatment allocation, are the gold-standard, as expectations from both parties can lead to over-estimation of effects (Colagiuri 2010).

Perhaps as a result of this, expectancy effects have often been conceptualised as nuisances which serve no purpose except needing to be controlled (Burke and Blumberger 2021). Nevertheless, evidence is now pointing towards a more nuanced picture, as research on expectancy has delineated the phenomenon as an observable neurobiological effect which may be responsible, at least in part, for the improvements observed in licenced treatments, including, for example, antidepressants (Burke and Blumberger 2021).

Expectancy can be influenced by a wide range of factors and is a feature common to all disorders across all of medicine (Zion and Crum 2018). Outside of clinical trials, expectancy is responsible for a significant proportion of medication-induced change in many disorders, including those which lie outside of psychiatry; for example, in one trial of deep brain stimulation in Parkinson’s disease, being unblinded as to the on–off status of stimulation significantly increased or decreased motor symptoms (Mercado et al. 2006). In the real world, as previous authors have put it, ‘patients always expect something out of the treatments they search for, and therapeutic alliance and placebo effects are always part of the process’ (Schenberg 2021). There are some who have called for the direct leveraging of expectancy effects when treating neuropsychiatric disorders (van Osch et al. 2017; Burke et al. 2019; Rommelfanger 2013).

Expectancy is formed at least in part from internalised information people receive about their disorder or treatments from external sources. For example, if I read an article that suggests this pill will be effective for my disorder, I might be more likely to experience symptom resolution than if I had not read the article. This is the case for all medications, including placebo medications (Fillmore and Vogel-Sprott 1992), and even when people are explicity told they are taking an inert substance (an ‘open-label’ placebo) (Kaptchuk and Miller 2018).

External influence on expectancy is particularly relevant to participants in psychedelic trials, many of whom are likely to have come across portrayals of psychedelics which claim the substances are proven to be highly effective (Aday et al. 2021). This is likely to lead to positive expectation (consciously, subconsciously or both) which is in turn likely to lead to improvements in symptoms after enrolment, regardless of the specific effects of the psychedelics. This phenomenon has not been explored in detail in modern clinical trials of psychedelics; however, a recent trial of microdosing of psychedelics showed that positive expectation of improvement predicted scores of well-being (Kaertner et al. 2021).

This effect also works conversely, in that positive expectations can fuel disappointment in trials of novel psychoactive medication when participants believe that they have been allocated to the placebo arm. If the participant had a degree of investment in receiving the active treatment (e.g. a belief that they would only get better with the active medication), then symptoms may worsen, either due to conscious disappointment or a version of the nocebo effect, thus leading to worse outcome in the placebo arm and subsequent enhancement of the treatment effect. This effect has been seen in parallel situations such as those allocated to waiting list control arms in trials of psychological therapy (Furukawa et al. 2014; Aday et al. 2021). Again, this situation is likely to be enhanced whilst there exists a significant hype around psychedelics.

**Fig. 1:**
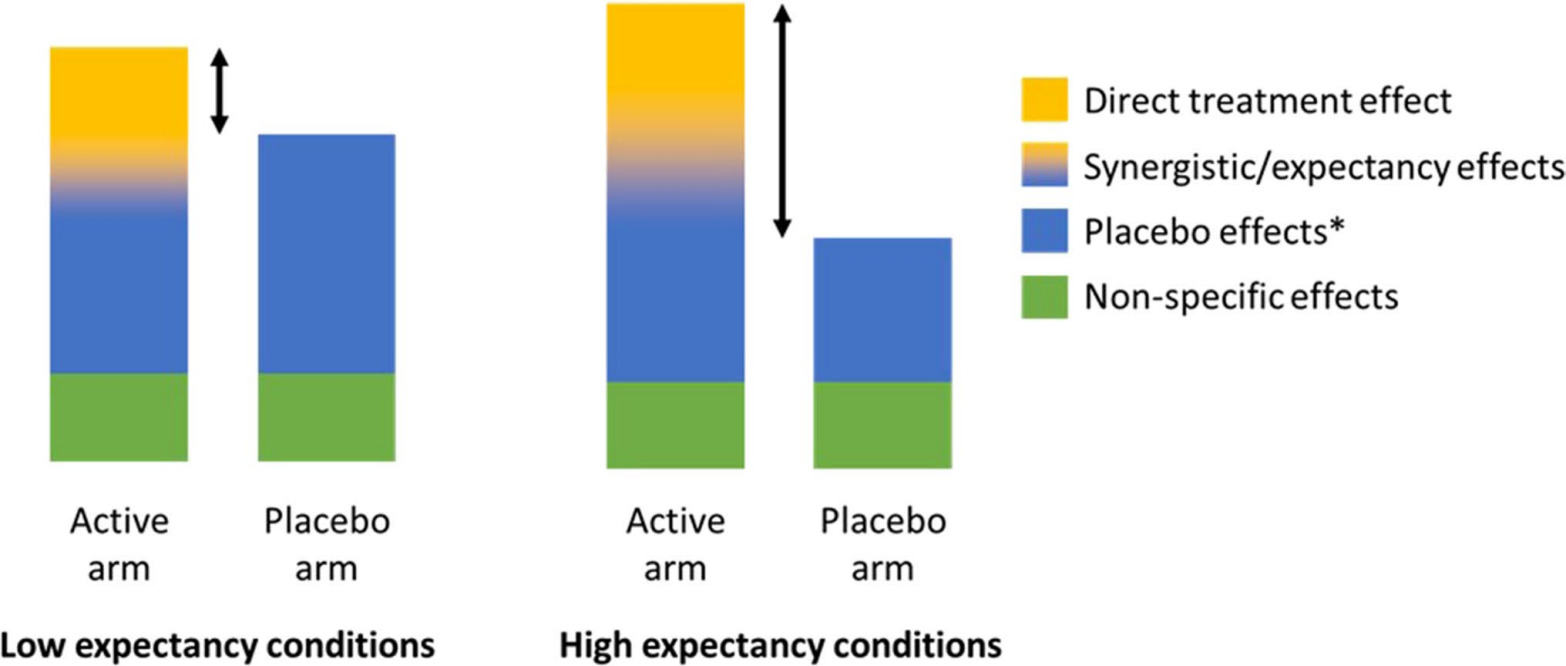
In this figure, we speculate how expectancy may influence results of placebo-controlled RCTs of psychedelics. The black arrows indicate relative treatment effects. The size of the direct treatment effect does not change; however, placebo and synergistic expectancy × treatment effects increase in the high expectancy group. If the participant had a degree of investment in receiving the active treatment (e.g. a belief that they would only get better with the active medication), then symptoms may worsen, either due to conscious disappointment or a version of the nocebo effect, thus leading to worse outcome in the placebo arm and subsequent enhancement of the treatment effect. The expectancy effects encompass those which arise from factors such as being enrolled in a trial and receiving psychological therapy (i.e., the non-medication effects). Non-specific effects include stochastic effects, regression to the mean, attenuation/elevation effect and Hawthorne effect (Burke et al. 2021)

In Fig. 1, adapted from (Burke et al. 2021), we speculate how overall effects may be modulated by expectancy in placebo-controlled RCTs of psychedelics. In conditions of low expectancy (i.e. the participant has a neutral stance on efficacy), there is some overlap between synergistic expectancy × treatment effects, but much of the difference in effect size arises from direct treatment effects. In conditions of high expectancy, where participants are convinced of a positive outcome, treatment effects are emphasised both from a larger synergistic expectancy × treatment response in the active arm, as well as a smaller expectancy effect (in other words, placebo minus nocebo effect) in the control arm, as participants are disappointed by their assessment of allocation to a placebo arm.

All medications are vulnerable to this external modification of expectation, however psychoactive substances are particularly so, and psychedelics even more so than the average psychoactive substance. The subjective experience of intoxication with psychoactive substances has been shown to be extremely sensitive to both individual expectation of what the trip will be like (the set) as well as environmental factors (the setting) (Carhart-Harris et al. 2018). The increased suggestibility associated with experiences under psychedelics suggests that particular caution must be taken when discussing their effects and effectiveness (J. A. Olson et al. 2020).

## Evidence from blinded randomised-controlled trials is sometimes imperfect

Previous authors have suggested that ‘the evidence for efficacy obtained from therapeutic RCTs of psychedelic drugs where masking has clearly failed falls short of the evidence obtained in other areas of medicine’ (Muthukumaraswamy et al. 2021). Although it is not difficult to find examples supporting this assertion, we argue that it may not be true in every sense. Although psychoactive medications certainly bring their unique challenges to blinding and expectation, they are not the only form of trial which faces issues with blinding; physiotherapy, surgery psychological therapies, and other psychoactive medications have all developed an evidence base despite not being a good fit for the blinded RCT model. We use these examples not as argument from precedent for hasty licencing of psychedelics — quite the contrary — but instead to suggest that although double-blinded RCTs indisputably produce the highest form of evidence when conducted appropriately, they may not be a perfect fit for interventions where expectancy can never be fully controlled.

As an example, blinding of allocation is very difficult in trials of physiotherapy. Although participants may nominally be blinded in a physiotherapy RCT, it is not difficult to imagine this blindedness being broken by the active participation (Boutron et al. 2004). As with all medical interventions, physiotherapeutic regimens should show evidence of efficacy from trials (ideally RCTs) prior to introduction into clinical practice, but positive outcomes from such trials are almost certainly inflated because of expectancy. This effect should, without fail, be taken into account. Nevertheless, in the absence of any feasible alternative means of generating higher-quality evidence, it remains a pragmatic consideration rather than a sole necessary or sufficient means of rejecting evidence.

On a related note, much of surgical practice is also based on evidence which has not been obtained from placebo-controlled randomised controlled trials. In trials of surgical procedures, blinding of surgeons is largely impossible, and it is difficult, but not impossible, to blind patients themselves. There are important situations where this is not the case; for example, recent evidence comparing routine orthopaedic procedures with sham surgery showed no between-group differences in pain management and quality of life (Louw et al. 2017). This is likely due to a complicated mix of expectancy-mediated improvement in the placebo group as well as tempered outcomes in the intervention group. In one review of the current state of surgical evidence which discussed the utility of modified RCTs to bolster the wider evidence base, the authors suggested that ‘it remains unclear if ornate blinding measures for surgical interventions are really justified by the gain of better evidence’ (Probst et al. 2016). A discussion of whether we can learn from surgery and employ sham psychedelic ‘procedures’ under anaesthesia research follows later.

Closer to home, trials of psychological therapies are well-recognised as presenting difficulties in terms of expectancy and blinding. The expectation that participants in psychological trials have about therapeutic gains is likely to influence the outcome, regardless of the effects of the specific therapy (Frank and Frank 1993). Indeed, positive early treatment outcome expectation is a robust predictor of treatment response across psychotherapy interventions (Constantino et al. 2018), which may be mediated by the quality of the therapeutic alliance (Vîslă et al. 2018). Furthermore, in clinical trials of psychological methods, participants are typically aware of the therapy they are receiving, and therapists are of course aware of which treatments they are delivering (Mataix-Cols and Andersson 2021)*.* Assessors may be blinded, which is the preferred state of things, but the issue of unblinding influencing participant expectancy remains. Reviews of the state of evidence have suggested that very few trials of psychological therapy adequately report blinding (Juul et al. 2021).

Modern clinical psychedelic research characteristically involves non-directive psychological support (Breeksema et al. 2020; Tai and Nielson, 2021; J. J. H. Rucker et al. 2018; J. Rucker et al. 2021), in which the therapeutic relationship between participant and therapists is of central importance (Richards 2015). This follows three phases: preparation, support during dosing and post-dose psychological integration (J. Rucker et al. 2021). Although participants in psychedelic trials receive the same type and frequency of therapy in each allocation arm, the content of the psychological therapy, particularly in the latter two phases, is likely to change based on participant and therapist assessment of treatment allocation (Gukasyan and Nayak 2021). Many modern trials of psychedelics thus share aspects integral to trials of psychological therapies, as opposed to being purely medication trials, and it is difficult (and arguably not useful) to wholly distinguish the effects of medication versus therapy (Gukasyan and Nayak 2021).

Finally, psychedelics are not unique in their ability to produce psychoactive effects. Other commonly prescribed medications with notable psychoactive effects have received licences, including benzodiazepines, opiates, methylphenidate, gabapentin and most recently nasal esketamine for treatment-resistant depression (Popova et al. 2019). Trials of such psychoactive medications have been shown to have high rates of correct allocation guesses from both participants and raters (Basoglu et al. 1997). Psychedelics, as with all of these treatments, should be subjected to rigorous scrutiny. Nevertheless, if we are to discount all treatments with psychoactive effects on the basis that they are impossible to blind, then we may be unknowingly restricting patient access to effective treatments (as the effective use of the above medications illustrates). This could be perceived as unethical as it would disproportionately discriminate against those who suffer from mental illness.

That is not to say that clinical trials should not strive to do better at incorporating expectancy and unblinding effects in both design and approval processes. In nasal esketamine studies, a bittering agent was added to the intranasal placebo to simulate the taste of the esketamine solution as an attempt to maintain blinding; however, as esketamine exhibits transient dissociative effects, blinding is unlikely to have been upheld (Popova et al. 2019). Despite no clear attempts to evaluate if blinding was maintained in the “double-blind” RCT data submitted to the regulatory authorities, esketamine has nonetheless received a license for treatment-resistant depression. The post-licencing debates on the effectiveness of esketamine illustrates the shortcomings of approving treatments based on standard-design RCT evidence alone, when a placebo ‘double-blinded’ RCT may not be the best fit.

## Staying pragmatic

Although RCTs provide the highest quality evidence for treatment efficacy in many cases, we have seen that they are not without limitations, and it follows that complementary forms of evidence should not be automatically dismissed if they are able to better adapt to issues of expectancy. RCTs have been criticised for their poor generalisability (Mulder et al. 2018; Nutt et al. 2020) and the false presumption that they are free from all sources of bias (Krauss 2018). Some authors have criticised the status of RCTs as the sole superior means of data generation for evidence bases (McCulloch et al. 2002; Nutt et al. 2020).

Forging a middle way, there may be other means of improving evidence from RCTs without dismissing them entirely, for example through ‘triangulating’ data from trials utilising different designs, for example observational cohort studies or pragmatic trials (Butler et al. 2021). The latter are trials which are broader in scope than a typical RCT and investigated whether treatments given in real-world circumstances have clinically meaningful effects (Purgato et al. 2015) and have been suggested by some as a good fit for psychedelic trials (Carhart-Harris et al. 2021).

Furthermore, RCTs rest on the assumption that the sole difference between treatment and placebo arms can be explained by the additive treatment effect. Despite this, there is emerging evidence that the outcome differences in placebo-controlled trials (of all treatments, not just psychedelics) are not simply the result of treatment minus expectancy effects; in other words, the treatment effect and expectancy effect are synergistic as opposed to additive. Research has shifted our understanding of placebo effects from a nuisance variable to a neurobiological phenomenon capable of modulating a range of neurotransmitter systems (including endogenous opioids, endocannabinoids, dopamine and oxytocin) and brain regions (Burke et al. 2021; L. Colloca and Barsky 2020).

While some neuroimaging work suggests effective placebo treatment induces changes in brain function that are distinct from response to antidepressants (Leuchter et al. 2002), other work has shown that placebos may activate similar areas of the brain as medications (Mayberg et al. 2002) In the case of neuromodulation for depression, there is further evidence that brain regions implicated in response to placebo overlap with treatment targets (Burke et al. 2021). The relationship between placebo and active treatment response is clearly more complicated than first assumed and is especially complex in the case of clinical psychedelic studies considering interactions between drug, psychotherapy process and potentially amplified placebo/nocebo effects in the case of unblinding.

## Previously proposed solutions to expectancy issues

Some of the proposed mechanisms whereby expectation in psychedelic trials may be reduced include assessing participant and researchers’ own ideas of expectation and treatment allocation. There is no currently no consensus on this as a process for clinical trials, partly given the difficulties in defining what exactly these questions are measuring (i.e. what are their hunches based on) (Schulz et al. 2010). Some have even suggested that testing for blinding may not, and often cannot, generate valid answers (Sackett 2007), and the CONSORT guidance on clinical trials do not recommend investigators measure expectancy (Moher et al. 2010). Furthermore, the perception of treatment allocation and the perception of treatment effects are somewhat distinguishable sets of expectations, both of which contribute to overall expectancy to unknown and likely changeable degrees (Colagiuri 2010). Nevertheless, given the unobtrusiveness of measuring expectancy and blinding and the important data it might generate, it seems very reasonable to recommend their inclusion to psychedelic trials.

Some have pointed out that to properly incorporate other suggestions on mitigating blinding in psychedelic trials, such as increasing the number of trials arms or comparators, would necessarily require large increases in the number of enrolled participants (Schenberg 2021) or lead to difficulties in accurate interpretation of results (Burke and Blumberger 2021). Another suggestion to try to mitigate expectancy has been to use methods of concealing the dose or treatment allocation, for example via deception. This could even go as far as a deceptive ‘psychedelic’ trial which is in fact entirely placebo. As has been pointed out by others, this raises ethical concerns which would likely preclude their use in clinical trials) (Schenberg 2021; Muthukumaraswamy et al. 2021).

There have also been suggestions that administering psychedelics under general anaesthesia would be an effective means of probing their context-independent antidepressant effects (D. E. Olson 2020). This seems like an interesting means of investigating medication response and disambiguating it from both the psychological therapy and the expectancy aspects. Nevertheless, caution should be exercised given that anaesthetics themselves may have antidepressant response (Tadler and Mickey 2018) or may interfere with psychedelics’ molecular mechanisms of action. Additionally, as we have seen, sham surgery under anaesthetic leads to positive outcomes, and therefore, this means of probing response to psychedelics may not be as informative as we would hope, given that the placebo procedure itself may be sufficient to lead to positive therapeutic outcomes.

This suggestion highlights a wider issue with psychedelic therapy as to whether the subjective effects are necessary for clinical improvement. As we have seen, it is often not difficult to guess treatment allocation in psychedelic trials given their profound effects on experiential consciousness. It is certainly an open question to the extent of how much the subjective effects themselves are necessary for improvement; however, given what we currently know about psychedelics and the integral role of supportive psychological therapy, the burden of proof probably lies with those who argue that the therapeutic effects are entirely independent of the subjective experience (Yaden and Griffiths 2020; D. E. Olson 2020). This is as true with psychedelics as it is with any pharmacological compound.

Placebo effects mediated by expectancy have shown to be particularly strong in trials of medication for depression (such as select serotonin reuptake inhibitors), and therefore, the effects of many antidepressant medications are likely inflated beyond a ‘true’ pharmacological effect (Rutherford et al. 2017). This raises important unanswered questions on how — if indeed we should at all — we might best prescribe antidepressants to increase and leverage expectancy. The argument could follow that regardless of how the improvement in suffering is achieved, the most important factor is the recovery itself.

If we accepted that improvement from psychedelics shared similar features, particularly as psychedelics are likely to affect the mechanism of expectancy formation, we must then think about what we should do in response. We would argue that the presence of substantial expectancy effect in itself would not be sufficient reason to cease research into the medical use of psychedelics, even if it is a not insignificant facet of symptom improvement. Nevertheless, this is a complex conundrum faced by clinicians and researchers, and the implications of any responses are potentially profound.

## Conclusions and recommendations

Researching psychedelics for neuropsychiatric disorders poses challenges to trial methodology and interpretation which, if not unique, are exaggerated. Expectancy effects and difficulties in blinding are commonplace across medicine, including in trials of physical interventions, psychotherapy and psychopharmacology. Beyond practical attempts to try and reduce expectancy and unblinding, psychedelic trials probably should not be held to different standards than other forms of clinical research, particularly as psychedelic trials share as much in common with other interventions which unintentionally unblind participants, such as psychological therapies, as they do standard pharmacological trials.

Although suggestions for reducing expectancy and unblinding in clinical trials, such as deception or incomplete disclosure of trial arms, are worthy of consideration, it is not clear whether they would be ethical, practical, generalisable or clinically meaningful. Instead, the field may have to come to terms with the notion that expectancy effects are essentially inextricable from the outcome. Taking lessons from other areas of healthcare research, non-RCT trials of psychedelics should remain a serious consideration to complement RCT evidence.

This is not to say that researchers should not make reasonable attempts to mitigate for expectancy effects. Placebo-controlled RCTs remain one of the best options for probing medical psychedelics and hence should be as well-designed as feasible. Active placebos, either low-dose psychedelics or alternative psychoactive medications (particularly those which induce a distinct but similarly profound psychoactive effect), likely provide a means of mitigating the expectancy effects; this may be particularly useful in those who are ‘psychedelic naïve’ (i.e. have no previous experience of psychedelics), as well as those who are active placebo naïve.

In trials of efficacy, independent and blinded external raters (ideally unaware of the nature of the intervention) should be used to assess symptoms, and patients should be advised not to share their hunch on treatment allocation to the assessor (Mataix-Cols and Andersson 2021). Psychedelic researchers should always be upfront about their attempts to mitigate for expectancy and where expectancy has not been controlled for.

Finally, one of the more effective means of reducing expectancy effects might be to reduce the excessive hype surrounding the psychedelic trials and premature assumptions of their place in clinical psychiatric practice. This requires equipoise from psychedelic researchers, as well as appropriate and balanced reporting of trials and their results from institutions and the media.

Even once these elements have been implemented, we may have to retain a certain sense of agnosticism about the results we find. It is unlikely ever to be possible to entirely disambiguate the effects of psychoactive drugs themselves from the expectancy effects that come with them. In attempting to do so, we have a duty to ensure we do not throw the therapeutic baby out with the bathwater. This seems particularly applicable to psychedelic therapy, where the drug is likely to affect the mechanism of expectancy formation.

King’s College London (KCL) receives grant funding for phase 1 and 2 trials with psilocybin, led by JR, from Compass Pathways Ltd. JR has attended trial-related meetings paid for by Compass Pathways Ltd. JR has undertaken paid consultancy work for Beckley-PsyTech and Clerkenwell Health. JR works for Sapphire Medical Clinics, a private medical cannabis clinic in the UK. MB and LJ work on the KCL psilocybin trials. The institutions mentioned had no influence over the inception, design, execution or publication of this work.
